# Tailoring diagnosis and treatment in symptomatic gallstone disease

**DOI:** 10.1093/bjs/znac154

**Published:** 2022-05-31

**Authors:** Carmen S S Latenstein, Philip R de Reuver

**Affiliations:** Department of Surgery, RadboudUMC, Nijmegen, the Netherlands; Department of Surgery, RadboudUMC, Nijmegen, the Netherlands

## Abstract

**Background:**

There is a lack of consensus in selecting patients who do or do not benefit from surgery when patients present with abdominal pain and gallbladder stones are present. This review aimed to give an overview of results from recent trials and available literature to improve treatment decisions in patients with uncomplicated cholecystolithiasis.

**Methods:**

First, an overview of different symptom criteria for laparoscopic cholecystectomy in patients with uncomplicated cholecystolithiasis is given, based on national and international guidelines. Second, treatment outcomes (absence of biliary colic, pain-free state, biliary and surgical complications) are summarized, with data from three clinical trials. Finally, personal advice for treatment decisions in patients with uncomplicated cholecystolithiasis is provided, based on recent trials, the available literature, and expert opinion.

**Results:**

This review describes different guidelines and criteria sets for uncomplicated cholecystolithiasis, provides an overview of outcomes after cholecystectomy, and advises on treatment decisions in patients with abdominal pain and gallbladder stones. After cholecystectomy, biliary colic is resolved in 95 per cent of patients. However, non-specific abdominal pain persists in 40 per cent. Irritable bowel syndrome and functional dyspepsia significantly increase the risk of persistent pain. Age, previous abdominal surgery, baseline pain score on a visual analogue scale, pain characteristics, nausea, and heartburn are part of the SUCCESS criteria, and are associated with clinically relevant pain reduction after gallbladder removal.

**Conclusion:**

The surgical community can now give more personalized advice on surgery to improve care for patients with abdominal pain and uncomplicated cholecystolithiasis.

## Teaser

The diagnostic evaluation and treatment of patients with abdominal pain and gallstones are subject to change. The present review has shown that the surgical community can improve patient selection for cholecystectomy, and recent research has increased awareness of the negative impact of concomitant abdominal disorders on pain reduction after cholecystectomy.

## Introduction

Worldwide, laparoscopic cholecystectomy is the most common abdominal surgical procedure, accounting for over 750 000 operations in the USA and approximately 70 000 operations in the UK each year^1,2^. A recent study^3^ of National Health Service data showed that the total number of cholecystectomies annually increased by 72.2 per cent between 2000 and 2019 (from 39 022 to 67 204). Although the number of laparoscopic cholecystectomies has increased over recent decades^4,5^, international guidelines^6–8^ have provided no reason to broaden the indication for surgery. The 5 per cent increase in the prevalence of gallbladder stones, from 15 to 20 per cent, does not explain the exponential increase in cholecystectomy numbers.

In Europe, approximately one-fifth of the population has gallbladder stones^9^. The majority of gallbladder stones consist primarily of cholesterol (over 90 per cent), whereas a minority are black (bilirubin) and brown (bacterial products) pigmented stones. Predisposing factors include obesity, female sex, diabetes mellitus, and hypertension, but only one in five develops symptoms^1^. Symptomatic patients mostly suffer from typical symptoms of biliary colic^10–12^. Frequent episodes of biliary colic or complicated gallstone disease (such as choledocholithiasis, cholecystitis, pancreatitis, or cholangitis) are accepted indications for cholecystectomy^6–8^. However, in most patients, gallstones remain without symptoms and are not the primary cause of abdominal pain. Patients with abdominal pain and uncomplicated symptomatic cholecystolithiasis are confronted with two options: wait and see or laparoscopic cholecystectomy. However, there is lack of consensus regarding selection of those who do or do not benefit from surgery, as 10–40 per cent of patients have persistent abdominal pain after operation^6–8^. This provides the clinical equipoise to suggest an alternative strategy for better selection of patients for cholecystectomy^13–16^.

The present review aimed to provide an overview of different guidelines and criteria sets for uncomplicated cholecystolithiasis; to summarize outcomes after cholecystectomy; and to give personalized advice on treatment decisions in patients with symptomatic cholecystolithiasis.

## Current primary care for patients with uncomplicated cholecystolithiasis

In the UK, approximately 2.3 per cent of the primary care population presents with abdominal pain each year^17^. Identifying the true aetiology of abdominal pain is the main challenge for several reasons. Most patients with cholecystolithiasis do not develop symptoms^1^. Only 60 per cent of patients in primary care with abdominal pain and a suspected diagnosis of gallbladder stones appear to have stones confirmed on radiological imaging^18^. Even with the diagnosis of gallstones, there is no definite correlation with abdominal pain^14,19^.

**Fig. 1:**
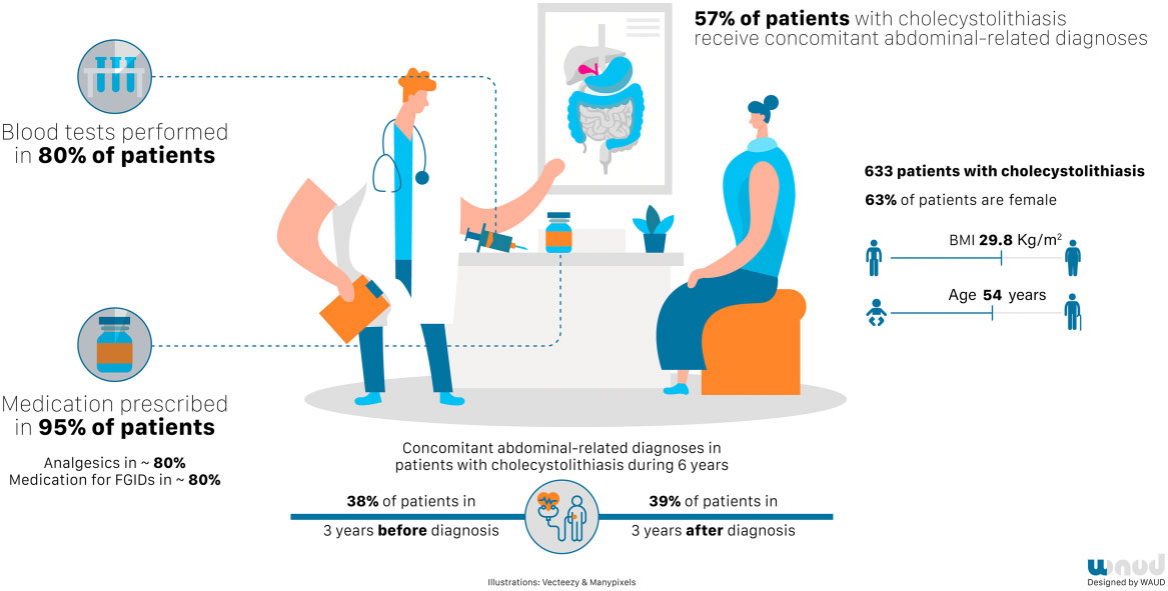
Typical patient with gallbladder stones in primary care setting Registry data from 633 patients^20^. FGID, functional gastrointestinal disorder.

The authors^20^ recently undertook a primary care analysis to explore general practitioner (GP) management of patients with gallstones in order to assess concomitant gastrointestinal diagnoses for pain. This study showed that 360 of 633 included patients with gallstones (57 per cent) were also diagnosed with another gastrointestinal disorder in the years before and after the diagnosis of symptomatic gallstone disease. Recorded International Classification of Primary Care codes were abdominal pain (31 per cent), stomach ache (14 per cent), constipation (11 per cent), and acid-related disease (10 per cent). Medication was prescribed to treat abdominal pain in 95 per cent of patients with gallstones. Most commonly prescribed medications included non-steroidal anti-inflammatory drugs (84 per cent), antacids (80 per cent), and analgesics (61 per cent). The patient characteristics of this cohort consulting a GP were similar to those of patients with gallstones consulting surgeons and gastroenterologists^14^, with a male : female ratio of 1 : 1.6, and a mean BMI of 30 kg/m^2^. *Figure 1* illustrates the characteristics, diagnostics, and treatment in primary care of patients with cholecystolithiasis.

Some 79 per cent of patients with gallstones in primary care are referred to secondary care. Patients were most often referred to the emergency department, surgeon or gastroenterologist (47.2, 42.5, and 10.3 per cent respectively). Ultimately, 79 per cent of all referred patients underwent cholecystectomy^20^. After cholecystectomy, more than half of the patients (52 per cent) returned to the GP with persistent abdominal pain. Specifically, if a patient was diagnosed with a concomitant abdominal disorder, their chance of returning to the GP with persistent abdominal pain was significantly increased compared with that of patients without such a diagnosis (51.9 *versus* 28.8 per cent; *P* < 0.001). This observation is consistent with a study^18^ in a general practice of 107 patients with gallstones in whom neither biliary colic nor another abdominal complaint was consistently related to gallstones. In the authors’ experience, it is pivotal that a GP manages the expectations of patients with regard to treatment outcomes early in the healthcare trajectory. Once a patient has been referred to a surgeon, implying a relationship between the presence of gallstones and pain, it is hard to convince a patient that a wait-and-see policy is more appropriate.

## Symptom criteria in patients with uncomplicated cholecystolithiasis

Quality assessment of 14 international guidelines on cholecystolithiasis showed that only five guidelines were suitable for clinical practice, as most recommendations were based on a low level of evidence^21^. For example, the definition of symptomatic gallstone disease and the indication for cholecystectomy are poorly described. Most guidelines advocate cholecystectomy in patients with symptomatic cholecystolithiasis or biliary colic^6,7^. The issue is, however, that both conditions are ill defined.

*Table 1* summarizes guidelines and criteria sets for symptomatic cholecystolithiasis. Most guidelines refer to the Rome criteria to define symptomatic gallstone disease: pain in severe attacks, in the epigastrium or upper right quadrant, and lasting 15–30 min or longer^10–12^. The Association of Upper Gastrointestinal Surgeons of Great Britain and Ireland commission guide^22^ from 2016 slightly modified these criteria to epigastric or upper right abdominal pain, frequently with radiation to the back, lasting from several minutes to hours, and often occurring during the night. The Society for Surgery of the Alimentary Tract^23^ has similar criteria (temporary epigastric or upper right abdominal pain, radiation to the right flank or back, and nausea), whereas the European Association for the Study of the Liver^6^ has reported more than 10 criteria. The various symptoms and criteria reported in these guidelines illustrate the lack of consensus among healthcare professionals.

**Table 1:** Guidelines and criteria for uncomplicated cholecystolithiasis

Guideline/criteria	Symptoms
**Association of Upper Gastrointestinal Surgeons of Great Britain and Ireland: Commissioning Guide Gallstone Disease (2016)**	Epigastric or right upper abdominal pain
	Frequently radiating to back
	Lasting for several minutes to hours
	(Often occurring at night)
**Society for Surgery of the Alimentary Tract: Treatment of Gallstone and Gallbladder Disease (2007)**	Temporary epigastric or right upper abdominal pain
	Radiates to the right flank or back
	Nausea
**American Academy of Family Physicians: Management of Gallstones (2005)**	Steady, non-paroxysmal pain
	Pain rapidly increases in intensity then plateaus
	Pain lasts 4–6 h
	Pain occasionally radiates to right subscapular area
**European Association for the Study of the Liver: Clinical Practice Guidelines on the Prevention, Diagnosis and Treatment of Gallstones (2016)**	Biliary colic
	Radiating pain
	Use of analgesics
	Nausea
	Vomiting
	Pain is severe and begins abruptly or increases progressively in intensity before stabilizing
	Irregular periodicity of pain
	Onset approximately 1 h after meals
	Onset during evening or at night
	Awakening patient from sleep
	Duration of more than 1 h
**German Society for Digestive and Metabolic Diseases: Guidelines for Diagnosis and Treatment of Gallstones (2007)**	Severe pain attacks
	Pain lasting 15–30 min or longer
	Pain located in epigastrium or right upper quadrant
	Nausea
	Vomiting
**Dutch Association of Surgery: Gallstone Disease (2016)**	Severe pain attacks
	Pain lasting 15–30 min or longer
	Pain located in epigastrium or right upper quadrant
**Rome criteria** ^10^	Severe pain attacks
	Pain lasting 15–30 min or longer
	Pain located in epigastrium or right upper quadrant
**SECURE criteria** ^14^	Severe pain attacks
	Pain lasting 15–30 min or longer
	Pain located in epigastrium or right upper quadrant
	Pain radiating to back
	Positive pain response to simple analgesics
**SUCCESS criteria** ^24^	Older patients
	No history of abdominal surgery
	Increased baseline pain score on VAS
	Pain radiating to back
	Positive pain response to simple analgesics
	Nausea
	Without heartburn

VAS, visual analogue scale.

This variation in criteria for surgery was one of the reasons for initiating the SECURE trial, which tested one set of symptom criteria^14^. The study randomized patients with gallstones and abdominal pain between usual care (diagnostics and treatment based on hospital standard care) and a restrictive strategy. In the restrictive arm of the study, cholecystectomy was advised only for patients who had all five prespecified symptoms based on the Rome III criteria and on predictors identified during a previous clinical study: severe pain in attacks, pain in the upper right quadrant or epigastrium, pain lasting 15–30 min or longer, pain radiating to the back, and positive response to simple analgesics^10–12,25^. The primary aim of the SECURE trial was to assess the percentage of patients who were pain-free after 1 year of follow-up. The trial demonstrated a 7 per cent higher cholecystectomy rate in the usual-care arm (75 per cent of 537 patients) compared with the restrictive arm (68 per cent of 530 patients) (*P* = 0.005). Despite more cholecystectomies, the usual-care strategy resulted in more patients who were pain-free after 12 months of follow-up (60 *versus* 56 per cent respectively; *P* = 0.316). Rates of gallstone-related complications were similar in the two groups: 8 per cent in the usual-care and 7 per cent in the restrictive arm (*P* = 0.155). The SECURE trial^14^ also illustrated the limited validity of Rome III criteria regarding the indication for cholecystectomy in patients with cholecystolithiasis; 35 per cent of patients with typical biliary colic (by Rome definition) reported persistent gastrointestinal symptoms after surgery.

## Outcome of cholecystectomy and cause of persistent abdominal pain

*Table 2* summarizes patient characteristics and preoperative symptoms in patients with uncomplicated cholecystolithiasis. The data are based on three clinical trials (SECURE, PERFECT, and SUCCESS), which included patients between 2014 and 2019 in 30 Dutch hospitals.

**Table 2:** Patient characteristics and preoperative symptoms in patients with uncomplicated cholecystolithiasis participating in clinical trials at RadboudUMC

	No. of patients (*n* = 1962)
**Patient characteristics**	
Age (years)*	50.3
Female sex (%)	73.6
BMI (kg/m^2^)*	27.6
History of abdominal surgery (%)	37.6
**Preoperative symptoms**	
VAS pain score*	7.7
Biliary colic (%)‡	66.6
Difficulty defaecating (%)	18.6
Diarrhoea (%)	16.2
Heartburn (%)	28.4
Abdominal bloating (%)	45.0
**Preoperative concomitant disorders**†	
Functional dyspepsia (%)	26.7
IBS (%)	17.2
Functional dyspepsia and/or IBS (%)	34.9

* Median value. Data based on patients included in the SECURE trial (1067), PERFECT trial (401), and SUCCESS trial (494); †based only on PERFECT trial. ‡According to Rome criteria. VAS, visual analogue scale; IBS irritable bowel syndrome.

**Fig. 2:**
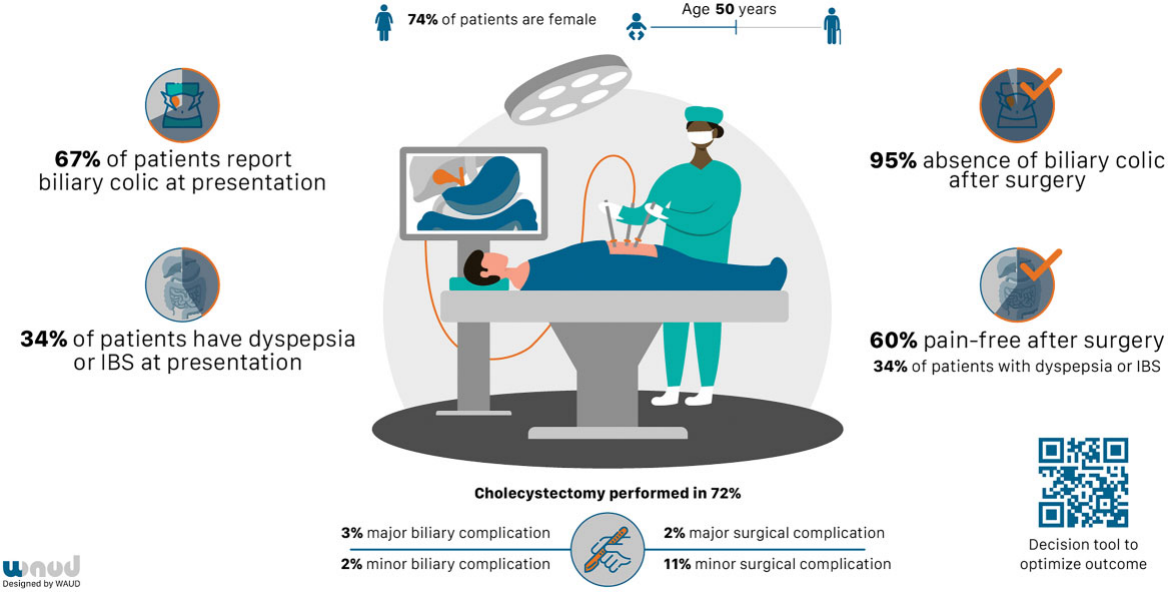
Patients with uncomplicated cholecystolithiasis at the surgical outpatient clinic Registry data based on the SECURE, PERFECT, and SUCCESS trials (total 1962 patients). IBS, irritable bowel syndrome.

The PERFECT^26^ and SUCCESS^24^ trials showed that laparoscopic cholecystectomy effectively resolved biliary colic. However, both trials confirmed that persistent abdominal pain after cholecystectomy is present in 40 per cent of patients^13–15^. Several causes of persistent abdominal pain after surgery have been identified. The literature suggests that several gastrointestinal symptoms originating from disease aetiologies other than gallstones are responsible for the development of postcholecystectomy pain^27,28^. Functional dyspepsia (FD) and irritable bowel syndrome (IBS) have a prevalence of 20–30 and 20 per cent respectively in the primary care population. Both are causes of pain with similar pain characteristics^1,29,30^. These conditions generally involve similar population groups: mainly middle-aged women who are overweight. These similarities complicate diagnosis of the condition responsible for a patient’s complaints and, as a result, potentially lead to abdominal pain being wrongfully attributed to gallstones and therefore influencing the choice of treatment (cholecystectomy)^27,28,31^. *Figure 2* is an infographic illustrating the characteristics and preoperative symptoms of patients with uncomplicated cholecystolithiasis included in the SECURE, PERFECT, and SUCCESS trials. The figure illustrates the percentage of patients with persistence of pain, and the impact of the concurrent presence of FD and IBS on postoperative pain relief. Finally, it shows that biliary complications occurred in 5 per cent of patients: 2 per cent minor (emergency department visit with biliary colic) and 3 per cent major (such as cholecystitis, cholangitis, or pancreatitis) biliary complications. Surgical complications occurred in 11 per cent and 2 per cent of patients underwent treatment for a major complication.

The PERFECT trial^26^ was initiated to evaluate the prevalence of FD and IBS in patients with gallbladder stones, and to explore the relationship between the concomitant presence of FD or IBS and patient-reported outcomes in terms of pain relief after laparoscopic cholecystectomy. This trial analysed 401 patients with symptomatic gallbladder stones (eligible for surgery). Of these patients, 34.9 per cent met the criteria for FD and/or IBS. A similar proportion of patients in the FD and/or IBS group and in the group without FD and/or IBS underwent surgery (74 *versus* 76 per cent respectively; *P* = 0.72). After cholecystectomy, 57 per cent of patients were pain-free; the difference between patients with and without FD and/or IBS was significant (41 *versus* 64 per cent respectively; *P* < 0.001). There was no significant difference between groups in the presence of biliary colic after surgery (5 *versus* 9 per cent; *P* = 0.22).

A previous prospective cohort study^19^ in the USA, focusing on identification of symptoms associated with upper abdominal pain relief after surgery, documented similar findings. Among 1008 patients who underwent cholecystectomy, only 59 per cent reported absence of upper abdominal pain 12 months after surgery. Preoperative pain characteristics (frequency, onset, duration, or timing) and presence of concomitant gastrointestinal symptoms or disorders (changed bowel pattern, nausea, bloating, gastro-oesophageal reflux disease, and/or IBS) predicted the odds of pain relief after cholecystectomy.

## Watchful waiting *versus* surgical treatment

In 2002, the first randomized study^32^ was performed that compared observation *versus* surgical intervention in patients with uncomplicated cholecystolithiasis. Sixty-eight patients were randomized to cholecystectomy and 69 to observation. During the 67 months of follow-up, 60 patients (88 per cent) and 35 patients (51 per cent) respectively eventually underwent cholecystectomy. No difference in admissions for pain attacks or gallstone-related complications, such as acute cholecystitis, choledocholithiasis or acute pancreatitis, was observed. Eight of 95 patients who underwent cholecystectomy developed a major complication. There were no differences between the groups in either quality-of-life or pain scores. In 2011, Schmidt and colleagues^33^ reported 14-year follow-up data, which showed that almost no operations were performed beyond 5 years in either group. Biliary complications occurred in 3 of 69 patients (4 per cent) randomized to observation; 1 of 68 patients (1 per cent) randomized to surgery had biliary pancreatitis before operation (*P* = 0.298). The authors concluded that, when the symptoms are intolerable, cholecystectomy is advised in patients with uncomplicated, symptomatic, gallbladder stones. However, conservative treatment is also an option, with a minimal risk of development of cholecystitis, cholangitis or pancreatitis.

The C-GALL trial^34^ is randomizing patients with symptomatic gallstones between surgical or conservative treatment. The investigators have hypothesized that there will be no difference in quality of life between conservative management and surgery after follow-up of 18 months. The study protocol includes all patients with radiologically confirmed gallbladder stones regardless of the severity and frequency of symptoms. This trial will investigate the effectiveness of surgical *versus* conservative treatment.

In the restrictive arm of the SECURE trial^14^, 171 patients did not undergo cholecystectomy. This group had characteristics (such as age, sex, and BMI) similar to those of the group that underwent cholecystectomy. However, these patients less frequently reported biliary colic, and had lower pain scores. As noted previously, biliary complications were similar between usual-care and restrictive strategies. The surgeon’s dogma of gallbladder removal to prevent biliary complications does not stand for all patients as the risk of developing biliary pancreatitis is below 2 per cent. Factors associated with biliary complications in patients with gastrointestinal pain and gallbladder stones should be investigated further. The 5-year follow-up of the Dutch trials will elucidate the rate of biliary complications in patients being treated according to a wait-and-see strategy.

## Choosing wisely

A shared decision regarding cholecystectomy between physician and patient is crucial, and most important for patients with concomitant FD and/or IBS. The patient should be aware of the high chance of persistent symptoms (divided into biliary colic and other non-biliary symptoms) after cholecystectomy, especially in patients with gallstones and FD and/or IBS.

Based on the available literature, the authors advise that the doctor should focus on patient characteristics, pain characteristics, and symptom severity during the first outpatient clinic visit. According to these pain characteristics and symptom severity, physicians should make the distinction between biliary colic and functional gastrointestinal symptoms. Cholecystectomy is the preferred treatment. However, when the most important symptoms match those of a functional abdominal disease, an evaluation after approximately 3 weeks is advised. In the meantime, the patient should register their symptoms. If, at the time of the evaluation, functional abdominal symptoms appear to be the main problem, these should be treated first. Expectant management is considered safe as the complication rate (development of cholecystitis, pancreatitis, or cholangitis) is very low.

**Fig. 3:**
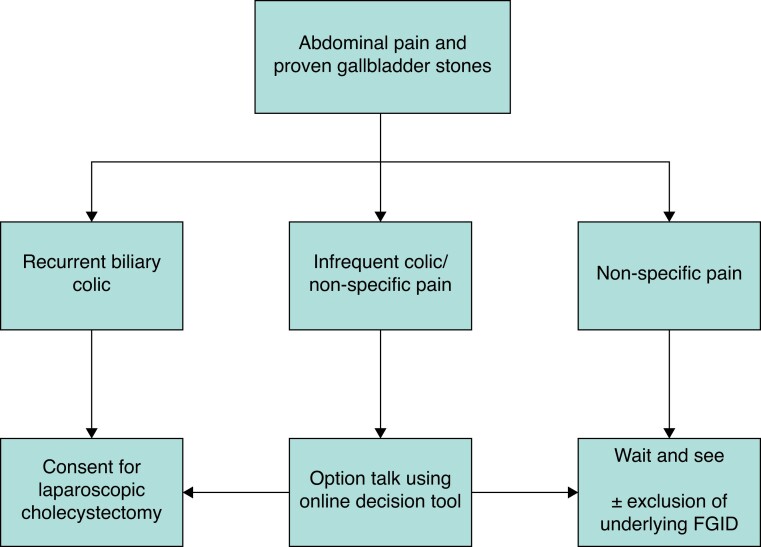
Decision tree for patient with uncomplicated gallstone disease at the surgical outpatient clinic FGID, functional gastrointestinal disorder.

In addition to this approach, a criteria set with patient characteristics and preoperative symptoms is of high value in improving treatment decision-making. In the SUCCESS trial^24^, a criteria set was developed and validated to allow selection of patients with uncomplicated gallstone disease for surgery based on prediction of becoming pain-free or achieving a relevant reduction in pain. The SUCCESS cohort provided the data for development of the criteria set: 494 patients with radiologically confirmed gallbladder stones, gastrointestinal pain symptoms, and a referral to the surgery department of the hospital. The criteria set comprised: older patients, with a high baseline pain score, no history of abdominal surgery, radiation of pain to the back, nausea, effective result after simple painkillers, and no symptoms of heartburn. Patients with these characteristics/symptoms more frequently had a relevant reduction in pain after gallbladder removal. External validation of the SUCCESS criteria in the SECURE data indicated good distinction between patients with and without a relevant reduction in pain. For daily clinical use, an online tool was developed, which can be accessed easily via the QR code in *Fig. 2* and at https://gallbladderresearch.shinyapps.io/SUCCESS/. To tailor treatment for individual patients, *Fig. 3* illustrates a decision tree. When patients with proven gallbladder stones complain of repeated biliary colic, this is considered to represent symptomatic gallstone disease, and cholecystectomy is advised in shared decision-making with the patient. An option talk is advocated for patients who report abdominal pain reminiscent of, but not completely typical of, biliary colic (33 per cent of patients) (*Table 2*). Use of the online decision tool is advised to predict the probability of a relevant pain reduction after surgery during this option talk with such patients. For example, for a 57-year-old patient, with a pain score of 7 and no history of abdominal surgery, absence of pain radiating to the back, a positive effect of simple painkillers, reporting nausea and heartburn, the predicted probability of a relevant pain reduction is 0.45 (95 per cent c.i. 0.23–0.70). Finally, an expectant policy is justified for patients with non-specific biliary pain or noted to have functional gastrointestinal disease. Other diagnoses must be considered in patients with atypical abdominal pain and, if symptoms remain, referral to the gastroenterology department or GP could be considered.

## Conclusion

The diagnostic evaluation and treatment of patients with gallbladder stones and abdominal pain are subject to change. The symptom criteria for symptomatic cholecystolithiasis differ across international guidelines. Although biliary colic is frequently resolved after cholecystectomy, non-biliary symptoms persist in up to 40 per cent of patients. This review guides surgeons on how to tailor diagnosis and treatment in patients with symptomatic cholecystolithiasis.

*Disclosure*. The authors declare no conflict of interest.

## Supplementary Material

znac154_Supplementary_Data
