# Supplementary material for: Tailoring diagnosis and treatment in symptomatic gallstone disease
Source: Br J Surg. 2022 May 31;109(9):832–8. doi: 10.1093/bjs/znac154 (PMC10364709; doi:10.1093/bjs/znac154)
Supplement: znac154_Supplementary_Data [file znac154_supplementary_data.docx]

**Tailoring diagnosis and treatment in symptomatic gallstone disease**

Carmen S.S. Latenstein^1^ MD PhD, Philip R. de Reuver^1^ MD PhD

^1^Department of Surgery, RadboudUMC, Nijmegen, the Netherlands

**Corresponding author:**P.R. de Reuver, surgeon, MD PhD
RadboudUMC
Department of Surgery
P.O. Box 9101
6500HB Nijmegen, The Netherlands
Email address: [philip.dereuver@radboudumc.nl](mailto:philip.dereuver@radboudumc.nl)
Telephone: +31 24 3617365

**Date of submission:** 7^th^ January 2022

**COI:** All authors have no COI or financial ties to disclose

**Funding:** This study did not receive any grant from funding agencies in the commercial, public, or not-for-profit sectors.

**Word count:** 2800

**References:** 34

**Keywords:** cholecystolithiasis; cholecystectomy; pain reduction; cost-effectiveness;

**ABSTRACT**

**Background:** There is a lack of consensus in selecting patients who do or do not benefit from surgery if patients present with abdominal pain and gallbladder stones are present. This review aimed to give an overview of results from recent trials and available literature to improve treatment decisions in patients with uncomplicated cholecystolithiasis.

**Methods:** First, an overview of different symptom criteria for a laparoscopic cholecystectomy in patient with uncomplicated cholecystolithiasis is given based on national and international guidelines. Second, treatment outcomes (absence of biliary colic, a pain-free state, biliary and surgical complications) are summarized with data from three clinical trials. Last, a personal advise for treatment decisions in patients with uncomplicated cholecystolithiasis is given based on recent trials, available literature, and expert opinion.

**Results:** The present review describes different guidelines and criteria sets for uncomplicated cholecystolithiasis, gives an overview of outcomes after cholecystectomy and advises on treatment decisions in patients with abdominal pain and gallbladder stones. After cholecystectomy, biliary colic is resolved in 95 per cent of patients. However, non-specific abdominal pain persists in 40 per cent. Irritable bowel syndrome and functional dyspepsia significantly increase the risk for persistent pain. Age, previous abdominal surgery, baseline VAS pain score, pain characteristics, nausea, and heartburn are part of the SUCCESS criteria and are associated with clinically relevant pain reduction after gallbladder removal.

**Conclusions:** The surgical community can now give a more personalised advice on surgery to improve care for patients with abdominal pain and uncomplicated cholecystolithiasis.

**TOC summary**

The diagnostic workup and treatment of patients with abdominal pain and gallstones are subject to change. The present review illustrates that our surgical community improves patient selection for cholecystectomy and recent research makes our community aware of the negative impact of concomitant abdominal-related disorders on pain reduction after cholecystectomy

**Introduction**

Worldwide, laparoscopic cholecystectomy is the most performed abdominal surgical procedure, accounting for over 750,000 operations in the United States (US) and approximately 70,000 operations in the United Kingdom (UK), every year.^1, 2^ A recent study in the National Health Service (NHS) data showed that the total number of cholecystectomies annually increased by 72.2 per cent, from 2000 to 2019 (from 39,022 to 67,204, respectively).^3^ And while the number of laparoscopic cholecystectomies increased over the past decades, international guidelines gave no reason to broaden the indication for surgery.^4-8^ The 5 per cent increase in the prevalence of gallbladder stones, from 15 to 20 per cent does not explain the exponential increase in cholecystectomy numbers.

In Europe, approximately one fifth of the population has gallbladder stones.^9^ The majority of gallbladder stones primarily consist primarily of cholesterol (>90 per cent), while a minority are black (bilirubin) and brown (bacterial products) pigmented stones. Predisposing factors include obesity, female gender, diabetes mellitus, or hypertension, but only one in five develops symptoms.^1^ Symptomatic patients mostly suffer from typical symptoms of biliary colic.^10-12^ Frequent episodes of biliary colic or complicated gallstone disease (e.g. choledocholithiasis, cholecystitis, pancreatitis, or cholangitis) are accepted indications for cholecystectomy.^6-8^ However, in most patients, gallstones remain without symptoms and are not the primary cause for the abdominal pain. Patients with abdominal pain and uncomplicated symptomatic cholecystolithiasis are confronted with two options: wait-and-see or a laparoscopic cholecystectomy. However, there is lack of consensus in selecting those who do or do no not benefit from surgery as 10-40 per cent of patients have persistent abdominal pain after surgery.^6-8^ This provides the clinical equipoise to suggest an alternative strategy on how to better select patients for cholecystectomy.^13-16^

The present review aims to first give an overview of different guidelines and criteria sets for uncomplicated cholecystolithiasis, second to summarize outcome after cholecystectomy and last to give a personalize advise on treatment decision in patients with symptomatic cholecystolithiasis.

**Current primary care for patients with uncomplicated cholecystolithiasis**

In the UK approximately 2.3 per cent of the primary care population presents with abdominal pain each year.^17^ Identifying the true aetiology of abdominal pain is the main challenge. First, most patients with cholecystolithiasis will not develop symptoms.^1^ Second, only 60 per cent of patients in primary care with abdominal pain and a suspected diagnosis of gallbladder stones appear to have stones confirmed on radiological imaging.^18^ Finally, even with the diagnosis of gallstones, there is no definite correlation with the abdominal pain.^14, 19^

We recently performed a primary care analysis to explore the GP (general practitioners’) management of gallstone patients to assess concomitant gastro-intestinal diagnoses for pain.^20^ This study showed that 360 of the 633 included gallstone patients (57 per cent) were also diagnosed with another gastro-intestinal related disorder in the years before and after the diagnosis of symptomatic gallstone disease. Recorded ICPC-codes (International Classification of Primary Care) were abdominal pain (31 per cent), stomach-ache (14 per cent), constipation (11 per cent), and acid-related disease (10 per cent). In 95 per cent of gallstone patients, medication was prescribed to treat abdominal pain. Most commonly prescribed medication included non-steroidal anti-inflammatory drugs (84 per cent), antacids (80 per cent), and analgesics (61 per cent). The patient characteristics of this cohort consulting a GP are similar to gallstone patients consulting surgeons and gastroenterologists with a male:female ratio of 1:1.6, and a mean BMI of 30kg/m^2^.^14^ Figure 1 illustrates the characteristics, diagnostics, and treatment in primary care of patients with cholecystolithiasis.

Seventy-nine per cent of primary care patients with gallstones are referred to secondary care. Patients were most often referred to the emergency department, surgeon or gastroenterologist, in 47.2, 42.5, and 10.3 per cent, respectively. Ultimately, 79 per cent of all referred patients underwent cholecystectomy.^20^ After cholecystectomy, more than half of the patients (52 per cent) returned to their GP due to persistent abdominal pain. Specifically, if a patient was diagnosed with a concomitant abdominal-related disorders their chance of returning to the GP with persistent abdominal pain was significantly increased compared to patients without such a diagnosis (51.9 vs. 28.8 per cent, p<0 .001). Our observation is consistent with a study in a general practice in 107 gallstone patients in whom neither biliary colic nor another abdominal complaint was consistently related to gallstones.^18^ In our experience, it is pivotal that a general practitioner manages the expectations of patients on treatment outcomes early in the healthcare trajectory. Once a patient is referred to a surgeon, implying al relation between the presence of gallstones and pain, it is hard to convince a patient that a wait-and-see policy is more appropriate.

**Symptom criteria in patients with uncomplicated cholecystolithiasis**

Quality assessment of 14 international guidelines on cholecystolithiasis showed that only five guidelines were suitable for clinical practice, as most recommendations were based on a low level of evidence.^21^ For example, the definition of symptomatic gallstones disease and the indication for cholecystectomy are poorly described. Most guidelines advocate cholecystectomy in patients with symptomatic cholecystolithiasis or biliary colic.^6, 7^ The issue is however that both conditions are ill-defined.

Table 1 summarizes guidelines and criteria sets for symptomatic cholecystolithiasis. Most guidelines refer to the Rome-criteria to define symptomatic gallstone disease: pain in severe attacks, in epigastrium or upper right quadrant and 15–30 min or longer.^10-12^ The AUGIS commission guide (2016) slightly modified these criteria to epigastric or upper right abdominal pain, frequently with radiation to the back, for several min to hours, and often occurring during the night.^22^ The SSAT (Society for Surgery of Alimentary Tract) has similar criteria^23^; temporary epigastric or upper right abdominal pain, radiation to the right flank or back, and nausea, while the EASL (European Association for Study of the Liver) reports over 10 criteria.^6^ The various symptoms and criteria reported in these guidelines illustrate the lack of consensus among healthcare professionals. This variation in criteria for surgery was one of the reasons to initiate the SECURE-trial. One set of symptom criteria was tested in the SECURE-trial.^14^

This study randomized patients with gallstones and abdominal pain between usual-care (diagnostics and treatment were based on hospital standard care) and a restrictive-strategy. In the restrictive-arm of the study a cholecystectomy was only advised patients who suffered from all five pre-specified symptoms based on Rome-III criteria and on predictors of identified during a previous clinical study; severe pain in attacks, pain in the upper right quadrant or in the epigastrium, pain for 15-30 min or longer, pain radiating to the back, and positive response after simple analgesics.^10-12, 24^ The primary aim of the SECUREtrial was to assess the percentage of pain free patients after one year of follow-up. The SECURE trial demonstrated a 7 per cent higher cholecystectomy rate in the usual care arm (75 per cent of 537 patients) compared to the restrictive study arm (68 per cent of 530 patients) (p =0.005). Despite more cholecystectomies, the usual care strategy resulted in more pain free patients after 12 months of follow-up (60 vs. 56 per cent, respectively, p = 0.316). Gallstone related complications were similar between groups; 8 per cent in usual-care and 7 per cent in the restrictive-arm of the study, p = 0.155). The SECURE-trial also illustrated the limited validity of Rome-III criteria for the indication of a cholecystectomy in patients with cholecystolithiasis 35 per cent of patients with a typical biliary colic (by the definition of ROME) reported persistent gastrointestinal symptoms after surgery.^14^

**The outcome of cholecystectomy and cause of persistent abdominal pain**

Table 2 summarizes the patient characteristics and pre-operative symptoms in patients with uncomplicated cholecystolithiasis. Data is based on three clinical trials (SECURE-trial, PERFECT-trial, and SUCCESS-trial) including patients between 2014 and 2019 in 30 Dutch hospitals.

The PERFECT- and SUCCESS-trial showed that laparoscopic cholecystectomy effectively resolved biliary colics.^25, 26^ However, both trials confirmed that persistent abdominal pain after cholecystectomy is present in 40 per cent of patients.^13-15^ Several causes have been identified for persistent abdominal pain after surgery. Literature suggests that several gastrointestinal symptoms originating from disease aetiologies other than gallstones are responsible for the development of ‘post-cholecystectomy pain’.^27, 28^ Functional-dyspepsia (FD), or irritable-bowel-syndrome (IBS)have a prevalence of respectively 20-30, and 20 per cent in the primary care population. Both are causes of pain with similar pain characteristics.^1, 29, 30^ These conditions generally involve similar population groups, i.e. mainly middle-aged women with overweight . These similarities complicate diagnosing the right condition responsible for patients’ complaints, and as a result potentially leads to wrongfully attributing abdominal pain to gallstones and therefore influence the choice of treatment (i.e. cholecystectomy).^27, 28, 31^ Figure 2 is an infographic illustrating characteristics and pre-operative symptoms of patients with uncomplicated cholecystolithiasis included in the SECURE-, PERFECT-, and SUCCESS-trial. The figure illustrates the percentage of patients with the persistence of pain and the impact of concurrent presence of FD and IBS on post-operative pain relief. Finally, the infographic shows that biliary complications occurred in 5 per cent of patients; 2 per cent minor biliary complications (emergency department visit with biliary colic) and 3 per cent major biliary complications (e.g. cholecystitis, cholangitis, or pancreatitis). Surgical complications occurred in 11 per cent of patients and 2 per cent of patients underwent treatment for a major complication.

The PERFECT-trial was initiated to first, evaluate the prevalence of FD and IBS in patients with gallbladder stones and second, to explore the relation between the concomitant presence of FD or IBS and the patient reported outcomes in terms of pain relief after laparoscopic cholecystectomy.^25^ This trial analysed 401 patients with symptomatic gallbladder stones(eligible for surgery). Of the patients, 34.9 per cent met criteria for FD and/or IBS. A similar proportion of patients in the FD and/or IBS group as in the group without FD and/or IBS underwent surgery (74per cent in FD/IBS versus 76per cent in the group without FD and/or IBS, p=0.72. After cholecystectomy, 57 per cent was pain-free. The difference between patients with and without FD and/or IBS was significant (41 in FD and/or IBS patients versus 64 per cent in the group without FD and/or IBS, p<0.001). The presence of a biliary colic after surgery was not significant (5 per cent in the group with FD and/or IBS versus 9 per cent the group without FD and/or IBS, p = 0.22).

A previous prospective cohort study performed in the United States focussing on identifying symptoms associated with upper abdominal pain relief after surgery showed similar findings. In 1008 patients who underwent cholecystectomy, only 59 per cent of the patients had absence of upper abdominal pain after 12 months postoperatively. Pre-operative pain characteristics (frequency, onset, duration, or timing) and presence of concomitant gastrointestinal symptoms or disorders (changed bowel pattern, nausea, bloating, GERD, and/or IBS) predicted the odds for pain relief after cholecystectomy.^19^

**Watchful waiting versus surgical treatment**

In 2002, the first randomized study was performed which compared the two strategies ‘observation’ vs. ‘surgical intervention’ in patients with uncomplicated cholecystolithiasis.^32^ Sixty-eight patients were randomized to cholecystectomy and 69 to observation. During the 67 months follow-up, sixty patients (88 per cent) and 35 patients (51 per cent) eventually underwent cholecystectomy. No difference in admissions for pain-attacks or gallstone related complications like acute cholecystitis, choledocholithiasis or acute pancreatitis was observed. Eight of 95 patients with a cholecystectomy suffered from a major complication. Both the quality of life and the pain scores did not differ between the groups. In 2011 the authors reported the 14 years follow up and showed that almost no surgeries were performed beyond five years within both groups. In patients randomized to observation, biliary complications occurred in 4 per cent (3 out 69 patients), in patients randomized to surgery one out of 68 patients had a biliary pancreatitis pre-operatively (1.5 per cent), p=0.298. The authors concluded that when the symptoms are intolerable a cholecystectomy is the advised treatment in patients with uncomplicated, symptomatic, gallbladder stones. However, a conservative treatment is also an option, with a minimal risk to develop cholecystitis, cholangitis or pancreatitis.^33^

The C-GALL-trial randomizes patients with symptomatic gallstones between surgical or conservative treatment . The investigators hypothesize that there is, in terms of quality of life, no difference between conservative management and surgery after a follow-up of 18 months.^34^ The study protocol includes all patients with radiologically confirmed gallbladder stones regardless of the severity and frequency of the symptoms. This trial will investigate the effectiveness of surgical treatment compared to conservative treatment.

In the restrictive arm of the SECURE trial, 171 patients did not undergo a cholecystectomy. This group had similar patient characteristics such as age, sex and BMI as the group who underwent cholecystectomy.^14^ However, these patients reported less frequently biliary colic, and lower pain scores. As previously described, biliary complications were similar between usual-care and restrictive-strategy. The surgeon’s dogma of gallbladder removal to prevent biliary complications does not stand for all patients as the risk to develop biliary pancreatitis less than 2 per cent. Factors associated with biliary complications in patients with gastro-intestinal pain an gallbladder stones should further be investigated. The five-year follow-up of the Dutch trials will illustrate the rate of biliary complications in patients with a wait and see strategy.

**Choosing wisely**

A shared decision between physician and patient for a cholecystectomy is crucial and most important in patients with concomitant FD and/or IBS. The patient should be aware of the high chance of persistent symptoms (divided in biliary colic and other non-biliary symptoms) after cholecystectomy, especially in patients with gallstones and FD and/or IBS.

Based on the available literature, we advise that during the first outpatient clinic visit, the medical doctor should focus on patient characteristics, pain characteristics and symptom severity. Based on these pain characteristics and symptom severity physicians should make the distinction between biliary colic and functional gastro-intestinal symptoms. When the primarily symptom is a biliary colic a cholecystectomy is the preferred treatment. However, when most important symptoms match with a functional abdominal disease, an evaluation after approximately 3 weeks is advised. In the meantime, the patient should register their symptoms. If at the time of the evaluation, functional abdominal symptoms appear to be the main problem, these should be treated first. An expectative management is considered safe as the complication rate (development of cholecystitis, pancreatitis, or cholangitis) is very low.

In addition to this approach, a criteria set with patient characteristics and pre-operative symptoms is of high value to improve treatment decision. In the SUCCESS-trial, a criteria set is developed and validated, to make a selection of patients with uncomplicated gallstones disease for surgery based on a prediction to become pain free or achieve a relevant pain reduction.^26^ The SUCCESS-cohort provided the data for the development of the criteria set: 494 patients with radiologically confirmed gallbladder stones, gastro-intestinal pain symptoms, and a referral to the surgery department of the hospital. Analysis showed that the criteria set consisted of patients with higher age, with a high baseline pain score, without previously abdominal surgery, with radiation of pain to the back, with nausea, with effect of simple painkillers, and no symptoms of heartburn. Patients with these characteristics/symptoms had more frequently a relevant pain reduction after gallbladder removal. External validation of the SUCCESS-criteria in the SECURE-data indicated good distinction between the patients with and without a relevant reduction of pain. For daily clinical use, an online tool was developed. The tool is easily accessible via the QR code in Figure 2 and via [https://gallbladderresearch.shinyapps.io/SUCCESS/](https://eur02.safelinks.protection.outlook.com/?url=https%3A%2F%2Fgallbladderresearch.shinyapps.io%2FSUCCESS%2F&data=04%7C01%7CPhilip.deReuver%40radboudumc.nl%7C4f3f4713b87b4666102408d9979171c4%7Cb208fe69471e48c48d87025e9b9a157f%7C1%7C0%7C637707473199701807%7CUnknown%7CTWFpbGZsb3d8eyJWIjoiMC4wLjAwMDAiLCJQIjoiV2luMzIiLCJBTiI6Ik1haWwiLCJXVCI6Mn0%3D%7C1000&sdata=x1sfJUh7s6z%2FPRn9%2FKw%2BP7f0Slp57nTyzcLGZeAM3ks%3D&reserved=0). To tailor the treatment for individual patients, Figure 3 illustrates a decision tree. When patients with proven gallbladder stones complain of repeated biliary colics, this is considered as symptomatic gallstone disease cholecystectomy is advised in shared decision with the patient. We advocate an option talk in patients who report abdominal pain reminiscent of but not completely typical of biliary colic (33 per cent of patients, table 2). We advise using the online decision tool to predict the probability of a relevant pain reduction after surgery during this option-talk in this group of patients. For example, in a 57 years old patient, with a pain score of 7 and no history of abdominal surgery, no radiation of pain to the back, a positive effect of simple painkillers, reporting nausea and heartburn; This patient's predicted probability of a relevant pain reduction is 0.45 (0.23-0.70). Finally, an expective policy is justified for patients with non-specific biliary pain or labelled with functional gastrointestinal disease. Other diagnoses have to be considered in patients with atypical abdominal pain and if symptoms remain referral to the gastroenterology department or GP could be considered.

**Conclusion**

The diagnostic workup and treatment of patients with gallbladder stones and abdominal pain are subject to change. The present review illustrates that the symptom criteria for symptomatic cholecystolithiasis differ across international guidelines. Although biliary colic is frequently resolved after cholecystectomy, non-biliary symptoms persist in up to 40 per cent of patients. This review guides surgeons how to tailor diagnosis and treatment in patients with symptomatic cholecystolithiasis.

**Summary box**

- In primary care, more than 50 per cent of patients with ultrasound diagnosed gallbladder stones are diagnosed with concomitant abdominal-related disorders.
- Laparoscopic cholecystectomy resolves biliary colic in 95 per cent of patients, however non-specific abdominal pain persist in up to 40 per cent.
- Functional-dyspepsia and irritable-bowel-syndrome significantly increase the risk for persistent pain after laparoscopic cholecystectomy.
- Predictive factors for pain relief after cholecystectomy are older age, without previous surgery, pain characteristics and absence of functional gastrointestinal disorders.

**References**

1. Lammert F, Gurusamy K, Ko CW, Miquel JF, Mendez-Sanchez N, Portincasa P, et al. Gallstones. *Nat Rev Dis Primers* 2016;**2**: 16024.

2. Sinha S, Hofman D, Stoker DL, Friend PJ, Poloniecki JD, Thompson MM, et al. Epidemiological study of provision of cholecystectomy in England from 2000 to 2009: retrospective analysis of Hospital Episode Statistics. *Surg Endosc* 2013;**27**(1): 162-175.

3. Lunevicius R, Nzenwa IC, Mesri M. A nationwide analysis of gallbladder surgery in England between 2000 and 2019. *Surgery* 2021.

4. Legorreta AP, Silber JH, Costantino GN, Kobylinski RW, Zatz SL. Increased cholecystectomy rate after the introduction of laparoscopic cholecystectomy. *JAMA* 1993;**270**(12): 1429-1432.

5. Kang JY, Ellis C, Majeed A, Hoare J, Tinto A, Williamson RC, et al. Gallstones--an increasing problem: a study of hospital admissions in England between 1989/1990 and 1999/2000. *Aliment Pharmacol Ther* 2003;**17**(4): 561-569.

6. European Association for the Study of the Liver . Electronic address eee. EASL Clinical Practice Guidelines on the prevention, diagnosis and treatment of gallstones. *J Hepatol* 2016;**65**(1): 146-181.

7. Agresta F, Campanile FC, Vettoretto N, Silecchia G, Bergamini C, Maida P, et al. Laparoscopic cholecystectomy: consensus conference-based guidelines. *Langenbecks Arch Surg* 2015;**400**(4): 429-453.

8. Evidence-based guideline: Diagnostic and Treatment of gallstones. <https://richtlijnendatabase.nl/richtlijn/galsteenlijden>. [March 2020.

9. Aerts R, Penninckx F. The burden of gallstone disease in Europe. *Aliment Pharmacol Ther* 2003;**18 Suppl 3**: 49-53.

10. The epidemiology of gallstone disease in Rome, Italy. Part II. Factors associated with the disease. The Rome Group for Epidemiology and Prevention of Cholelithiasis (GREPCO). *Hepatology* 1988;**8**(4): 907-913.

11. Prevalence of gallstone disease in an Italian adult female population. Rome Group for the Epidemiology and Prevention of Cholelithiasis (GREPCO). *Am J Epidemiol* 1984;**119**(5): 796-805.

12. The epidemiology of gallstone disease in Rome, Italy. Part I. Prevalence data in men. The Rome Group for Epidemiology and Prevention of Cholelithiasis (GREPCO). *Hepatology* 1988;**8**(4): 904-906.

13. Lamberts MP, Den Oudsten BL, Gerritsen JJ, Roukema JA, Westert GP, Drenth JP, et al. Prospective multicentre cohort study of patient-reported outcomes after cholecystectomy for uncomplicated symptomatic cholecystolithiasis. *Br J Surg* 2015;**102**(11): 1402-1409.

14. van Dijk AH, Wennmacker SZ, de Reuver PR, Latenstein CSS, Buyne O, Donkervoort SC, et al. Restrictive strategy versus usual care for cholecystectomy in patients with gallstones and abdominal pain (SECURE): a multicentre, randomised, parallel-arm, non-inferiority trial. *Lancet* 2019;**393**(10188): 2322-2330.

15. Vetrhus M, Berhane T, Soreide O, Sondenaa K. Pain persists in many patients five years after removal of the gallbladder: observations from two randomized controlled trials of symptomatic, noncomplicated gallstone disease and acute cholecystitis. *J Gastrointest Surg* 2005;**9**(6): 826-831.

16. Escarce JJ, Chen W, Schwartz JS. Falling cholecystectomy thresholds since the introduction of laparoscopic cholecystectomy. *Jama* 1995;**273**(20): 1581-1585.

17. Wallander MA, Johansson S, Ruigomez A, Garcia Rodriguez LA. Unspecified abdominal pain in primary care: the role of gastrointestinal morbidity. *Int J Clin Pract* 2007;**61**(10): 1663-1670.

18. Berger MY, Olde Hartman TC, van der Velden JJ, Bohnen AM. Is biliary pain exclusively related to gallbladder stones? A controlled prospective study. *Br J Gen Pract* 2004;**54**(505): 574-579.

19. Thistle JL, Longstreth GF, Romero Y, Arora AS, Simonson JA, Diehl NN, et al. Factors that predict relief from upper abdominal pain after cholecystectomy. *Clin Gastroenterol Hepatol* 2011;**9**(10): 891-896.

20. Thunnissen FM, Drager LD, Braak B, Drenth JPH, van Laarhoven C, Schers HJ, et al. Healthcare utilisation of patients with cholecystolithiasis in primary care: a multipractice comparative analysis. *BMJ Open* 2021;**11**(11): e053188.

21. van Dijk AH, de Reuver PR, Besselink MG, van Laarhoven KJ, Harrison EM, Wigmore SJ, et al. Assessment of available evidence in the management of gallbladder and bile duct stones: a systematic review of international guidelines. *HPB (Oxford)* 2017;**19**(4): 297-309.

22. Commissioning guide Gallstone Disease. [www.rcseng.ac.uk](file:///Users/kjetilsoreidemacpro/Desktop/*BJS%20Editor%20Workfile/****BJS%202022/Manuscripts%20/BJS-0049%20gallstone%20surgery%20revised/www.rcseng.ac.uk) › rcs › standards-and-research › commissioning.

23. Treatment of gallstone and gallbladder disease. SSAT patient care guidelines. *J Gastrointest Surg* 2004;**8**(3): 363-364.

24. Berger MY, van der Velden JJ, Lijmer JG, de Kort H, Prins A, Bohnen AM. Abdominal symptoms: do they predict gallstones? A systematic review. *Scand J Gastroenterol* 2000;**35**(1): 70-76.

25. de Jong JJ, Latenstein CSS, Boerma D, Hazebroek EJ, Hirsch D, Heikens JT, et al. Functional Dyspepsia and Irritable Bowel Syndrome are Highly Prevalent in Patients With Gallstones and are Negatively Associated With Outcomes After Cholecystectomy: A Prospective, Multicentre, Observational Study (PERFECT - Trial). *Ann Surg* 2020.

26. Latenstein CSS, Hannink G, van der Bilt JDW, Donkervoort SC, Eijsbouts QAJ, Heisterkamp J, et al. A Clinical Decision Tool for Selection of Patients With Symptomatic Cholelithiasis for Cholecystectomy Based on Reduction of Pain and a Pain-Free State Following Surgery. *JAMA Surg* 2021;**156**(10): e213706.

27. Latenstein CSS, Wennmacker SZ, de Jong JJ, van Laarhoven C, Drenth JPH, de Reuver PR. Etiologies of Long-Term Postcholecystectomy Symptoms: A Systematic Review. *Gastroenterol Res Pract* 2019;**2019**: 4278373.

28. Latenstein CSS, de Jong JJ, Eppink JJ, Lantinga MA, van Laarhoven C, de Reuver PR, et al. Prevalence of dyspepsia in patients with cholecystolithiasis: a systematic review and meta-analysis. *Eur J Gastroenterol Hepatol* 2019;**31**(8): 928-934.

29. Tack J, Talley NJ, Camilleri M, Holtmann G, Hu P, Malagelada JR, et al. Functional gastroduodenal disorders. *Gastroenterology* 2006;**130**(5): 1466-1479.

30. Sperber AD, Dumitrascu D, Fukudo S, Gerson C, Ghoshal UC, Gwee KA, et al. The global prevalence of IBS in adults remains elusive due to the heterogeneity of studies: a Rome Foundation working team literature review. *Gut* 2017;**66**(6): 1075-1082.

31. Schmidt M, Sondenaa K, Dumot JA, Rosenblatt S, Hausken T, Ramnefjell M, et al. Post-cholecystectomy symptoms were caused by persistence of a functional gastrointestinal disorder. *World J Gastroenterol* 2012;**18**(12): 1365-1372.

32. Vetrhus M, Søreide O, Solhaug JH, Nesvik I, Søndenaa K. Symptomatic, non-complicated gallbladder stone disease. Operation or observation? A randomized clinical study. *Scand J Gastroenterol* 2002;**37**(7): 834-839.

33. Schmidt M, Søndenaa K, Vetrhus M, Berhane T, Eide GE. A randomized controlled study of uncomplicated gallstone disease with a 14-year follow-up showed that operation was the preferred treatment. *Dig Surg* 2011;**28**(4): 270-276.

34. Ahmed I, Innes K, Brazzelli M, Gillies K, Newlands R, Avenell A, et al. Protocol for a randomised controlled trial comparing laparoscopic cholecystectomy with observation/conservative management for preventing recurrent symptoms and complications in adults with uncomplicated symptomatic gallstones (C-Gall trial). *BMJ Open* 2021;**11**(3): e039781.

**FIGURE LEGENDS**

**Figure 1. The classis patient with gallbladder stones in primary care setting**

Registry data from 633 patients. *BMI*; body mass index
Published In *BMJ Open*, 2021. Thunnissen et al. Healthcare utilization of patients with cholecystolithiasis in primary care: a multi-practice comparative analysis.

**Figure 2. Patients with uncomplicated cholecystolithiasis at the surgical outpatient clinic**

Registry data based on the SECURE-trial, PERFECT-trial and SUCCESS-trial (total n=1962).
*IBS;* Irritable bowel syndrome

**Figure 3. A decision tree for patient with uncomplicated gallstone disease at the surgical outpatient clinic.**
*FGID* Functional gastrointestinal disorders
